# Supplementary material for: Are carbon emissions trading and green financial instruments synergistic? -Comprehensive quantitative research based on content analysis
Source: PLoS One. 2024 Mar 7;19(3):e0298601. doi: 10.1371/journal.pone.0298601 (PMC10919601; doi:10.1371/journal.pone.0298601)
Supplement: S1 Data — (ZIP) [file pone.0298601.s001.zip › Data/地级市Z_F工作报告.zip!/╜¡╬≈/╔╧╚─╩╨/└┤╫╘╣1⁄2╓┌║┼í╛┬φ┐╦╩2╛▌═°í┐_2019.docx]

各位代表：

又是一年交卷时。

现在，我代表市人民政府向大会报告工作，请予审议，并请各位政协委员和列席会议的同志提出意见。

一、同心干、用心答，答出攻坚克难、砥砺奋进的考试卷

习近平总书记指出，“时代是出卷人，我们是答卷人，人民是阅卷人。”过去的一年，面对复杂多变宏观环境和艰巨繁重改革发展任务的新考卷，我们在省委、省政府和市委的坚强领导下，全面贯彻习近平新时代中国特色社会主义思想和党的十九大精神，围绕稳增长、促改革、调结构、优生态、惠民生、防风险各项工作，同心应考、用心答题，迎难而上、排难而进，圆满完成了市四届人大三次会议确定的目标任务，经济运行呈现高质量跨越式发展的鲜明特征。

——“快”的势头强劲。生产总值2212.8亿元，增长9.0%，分别比年初预期目标和全省平均水平高0.5和0.3个百分点；财政总收入351.6亿元，增长10.3%，分别比预期和全省高3.3和0.2个百分点；规上工业增加值增长9.4%，分别比预期和全省高0.8和0.5个百分点；城镇居民人均可支配收入34656元，增长8.8%，分别比预期和全省高0.3和0.4个百分点；农村居民人均可支配收入13346元，增长9.6%，分别比预期和全省高0.6和0.4个百分点。

——“好”的质量彰显。三次产业结构由12.2:47.4:40.4调整为11.4:46.1:42.5，三产比重提高2.1个百分点。税收占财政总收入的比重达到78.3%，较上年提高1.7个百分点，创近6年来新高。金融总量迈上6000亿元台阶、达到6246.9亿元；信贷投放增幅达到21.3%，再居全省第一；存贷比74.8%，较上年提高6.2个百分点，创近11年来新高。

——“美”的风貌提升。城镇化率达到51.97%，较上年提高1.55个百分点。棚改工作获得国务院通报表扬。新建秀美乡村点4192个，横峰、信州、婺源、三清山提前两年完成“扫一遍”任务。全省农村工作会议暨新农村建设现场推进会、省市县三级重大项目推进动员大会、全省深入推进基层社会治理工作暨综治中心实体化建设现场会等大型会议相继在我市召开。

一年来，我们齐心协力“打硬仗”，精准发力“解难题”，铿锵有力“谱新篇”，主要做了以下工作。

（一）坚持发展为先，产业升级提质提速

新型工业加速壮大。规上工业企业净增127家，总数达到1430家。新增上饶经开区汽车产业集群、德兴黄金产业集群为省级重点工业产业集群。加速“两光一车”产业发展，晶科能源组件出货量占全球份额突破10%、达到12%；海康智慧物联网产业园、维真裸眼3D等项目顺利推进；吉利新能源、长安跨越正式开工，中汽瑞华、博能商用车顺利推进，汉腾二期、爱驰汽车整车下线。实施县域工业攻坚，各县（市、区）集中财力、集中要素重点发展3个以内的主导产业。全市开工建设标准厂房1162万平方米，已建成780万平方米，搭建了产业快速集聚的良好平台。

现代服务业提档升级。全域旅游协同发展。开工建设城东文化旅游综合体、城西“十里槠溪”国际生态旅游度假区、城南捷成梦都数字影视科技文化城等投资过百亿项目。出台进一步加快发展乡村旅游的意见，成功举办首届上饶乡村旅游文化节，余干黄金埠板桥、婺源紫阳马家、玉山岩瑞包溪、横峰莲荷夏阳、铅山鹅湖紫竹林、弋阳旭光江廖肖等一大批乡村旅游示范点陆续建成，新增3A级以上乡村旅游点93个、总数达到168个，婺源瑶湾获评全国5个乡村振兴旅游目的地之一。新增国家4A级景区5家，总数达到30家。设立优质旅游诚信理赔基金1000万元。全年接待游客1.8亿人次、旅游综合收入1820.6亿元，分别增长13.1%、22.9%。国际医疗旅游先行区和国家中医药健康旅游示范区建设进展顺利。国内最大的多能干细胞库和细胞制造中心投入运营，汉氏联合健康体检中心、医学检验中心、医美抗衰中心即将建成。成功举办国家中医药健康旅游建设经验交流暨标准研讨会、上饶国际干细胞应用与大健康产业发展高峰论坛。德兴、婺源获批国家中医药健康旅游示范基地。数字经济示范区建设步伐加快。华为云数据中心基本建成，文娱创意中心、滴滴上饶全国呼叫总部一期顺利运营，大数据科创城全面启动，江西阿里云大数据学院正式落户。成功举办首届中国赣菜美食节暨第二届饶帮菜美食文化节。上饶国际物流园加快建设。实施企业上市“映山红行动”，晶科电力IPO申请获国家证监会受理，耐普矿机、艾芬达卫浴等企业进入辅导备案阶段，28家企业入选江西省重点上市后备企业资源库。

现代农业稳步增效。严格落实粮食安全责任制，粮食产量70.2亿斤。深入落实“东西南北中”特色产业布局，新增马家柚、虾蟹、红芽芋、茶叶、蔬菜面积58.4万亩，总面积达到324.4万亩。完成农村土地流转238.4万亩，流转率达到45.8%。建成高标准农田56.5万亩。新引进农业项目46个，山东鲁花等知名企业相继落户。新增“三品一标”农产品62个，总数达到601个。新增铅山江天农业为国家级农业产业化龙头企业。德兴、广丰入围全国农村一二三产业融合发展先导区创建单位。

（二）坚持统筹协调，城乡建设绘声绘色

基础设施有力推进。昌景黄高速铁路、上饶至浦城高速公路、德州至上饶（皖赣界至婺源段）高速公路、鄱阳至余干高等级公路正式动工，上饶铁路西货站、上饶动车组存车场、信江八字嘴航电枢纽、双港航运枢纽顺利推进。三清山机场新增舟山、昆明、哈尔滨3个通航城市，旅客吞吐量达到37.5万人次。上饶500千伏输变电环网正式形成，园北、万花220千伏输变电工程全面开工。

宜居城市加快建设。中心城区天佑大道、稼轩大道、滨江西路、茶圣东路等21条主次干道建成通车。市规划展示馆、城建档案馆、博物馆顺利开馆，市老年人综合活动中心基本建成，市美术馆加快建设。大力实施“去杂乱、补短板”工程，拆除改造杂乱面积62.6万平方米，新建小公园10个，改造提升里弄小巷19条，布局实施智能停车项目9个、新增停车位6000个，启动建设菜市场4个、垃圾中转站4个。完成各类棚改50255套。高标准亮化城区主干道25公里、信江岸线7.5公里，山、水、桥、楼、树交相辉映、流光溢彩，立体演绎了上饶美轮美奂的夜景长卷。制定城镇“厕所革命”三年计划，新建改造城区公厕17个。全面启动国家“公交都市”创建工作，新增、优化公交线路18条。

乡村基础持续提升。拆除农村违章房、危旧房、废弃房290多万平方米，完成赣派民居改造4.3万栋。建成农村公路1548.7公里，婺源、横峰获评“四好农村路”全国示范县。实施危桥改造97座。推进水利项目184个，完成投资8.1亿元。农村集体资产清产核资完成97.3%，村集体经营性收入3万元以下的“空壳村”全面消除。积极稳妥推进绿色殡葬改革，规划公益性墓地2954个、建成2599个，规划安息堂88个、建成47个，全市火化率一举实现100%。

城乡管理日趋规范。全面实施《城市管理条例》《农村居民住房建设管理条例》，城乡管理步入法治化、规范化轨道。扎实推进城乡环境综合整治，大力开展“百日攻坚”净化行动，组织评选中心城区“最干净街道（乡镇）”，在信州、德兴、婺源启动生活垃圾分类试点，全市基本实现城乡环卫一体化。全国文明城市、国家卫生城市创建工作全面启动。

生态文明建设力度空前。实施国土绿化工程，完成造林14.8万亩，占省下达任务的111.2%。不断深化河长制，全面建立湖长制、林长制。狠抓中央环保督察反馈问题整改，广泛发动群众参与，全面摸排和解决环保突出问题。深入开展长江经济带“共抓大保护”攻坚行动，关停封堵入河排污口115个，关闭污染企业212家，新建污水管网70公里。集中开展打击电鱼网鸟专项整治行动，查处违法案件1100多起，收缴非法电渔具1.35万套。7个生活垃圾焚烧发电项目全面开工，其中万年项目建成投运。累计关停生猪养殖场917家，拆除黏土砖瓦窑700多座。婺源被国家生态环境部评为“绿水青山就是金山银山”实践创新基地。全市饮用水源达标率100%；主要河流断面水质达标率96.9%，提高3.3个百分点，圆满完成省委、省政府下达的消灭劣V类水任务。城区空气质量优良率91.8%，提高6.3个百分点；PM2.5浓度均值36微克/立方米，下降18.2%。

（三）坚持创新引领，改革开放向深向好

重点改革有序推进。市县机构改革基本完成。政务服务“一网、一门、一次”改革成效明显，市本级依申请类政务服务事项网上可办率达到100%，进驻办事大厅事项全部实现受、办一体化。国资国企改革扎实推进，新组建市医投集团、教投集团、水投集团、农垦集团，上投集团资产总额突破2000亿元，达到2251亿元，信用等级获评国内最高的3A级，负债率36.7%，较上年降低19.6个百分点。扎实推进商事制度改革，深入实施“三十九证合一”，大力推广应用电子营业执照，新增私营企业21086户，增长17%；新增个体工商户45724户，增长22%。创新存量用地开发利用模式，消化批而未用土地3.2万亩，批而未用率下降14个百分点。稳步推进县乡医疗服务一体化试点。在市本级6家公立医院开展编制备案试点。事业单位和市属国有企业公务用车制度改革全面完成。

开放合作持续深化。扎实开展主导产业龙头项目招大引强竞赛，引进达产后主营业务收入超30亿元龙头项目20个、超10亿元骨干项目22个。以饶商联合总会换届为契机，联动推进项目、资金、技术、总部、人才“五位一体”回归。实际利用外资12.5亿美元，增长9.5%；引进省外2000万元以上项目资金729.9亿元，增长11.1%。新增“走出去”企业7家。上饶—宁波“海铁联运天天班”稳定运行。上饶至哈萨克斯坦中欧班列顺利开行。

创新活力不断增强。玉山致远环保参与研发的技术创新成果荣获国家技术发明二等奖。国家高新技术企业新增169家，总数达到328家。组建新型研发机构5个，获批省级工程技术研究中心7个。启动国家光学镜头镜片产品质量检验中心建设。新增“江西名牌”企业24家。开展“招才引智全国行”系列活动，新引进国家“千人计划”专家5名，新认定高层次人才26名。选树“上饶工匠”50名，5人获评江西省“能工巧匠”。

（四）坚持靶向操作，民生改善有力有效

脱贫攻坚成效显著。紧扣“两不愁、三保障”总体目标，接续开展春季攻势、夏季整改、秋冬会战行动，横峰、上饶县高质量脱贫“摘帽”，183个贫困村退出，9.2291万贫困人口脱贫，贫困地区面貌和群众生产生活条件发生可喜变化。倾斜聚力支持鄱阳、余干脱贫攻坚。城镇贫困群众脱贫解困工作正式启动。

民生事业加快发展。完善就业创业服务体系，新增城镇就业6万人、转移农村劳动力8.5万人，分别完成年度目标的122.5%、118.7%。城镇登记失业率3.2%。参加城镇职工养老保险115.4万人，征缴基金83.6亿元，完成年度目标的152.8%。市中心城区一中新校区、三中、信美学校、叶挺学校、十五小、明叔小学、时乔小学、吉阳学校全面动工。余干、铅山、横峰、鄱阳高质量通过国家评估，全市域实现义务教育基本均衡。方志敏干部学院建成开学。江西婺源茶叶职业学院揭牌成立。上饶卫校新校区正式启用。市人民医院城北院区主体封顶，市妇幼保健院、市第二人民医院综合楼全面装修。计划生育工作连续三年蝉联全省第一。成功举办第二届上饶文化创意产业博览会。电影《信仰者》广受好评，鄱阳大鼓《晒秋》荣获中国曲艺牡丹奖文学奖，填补江西空白。成功承办第五届全国地方志优秀成果研讨会。成功举办上饶国际马拉松赛、玉山斯诺克世界公开赛等大型体育赛事39个。

社会治理有效加强。全面完成第十届村（居）委会换届选举工作。开展安全生产“十大专项整治”行动和“企业主体责任强化年”活动，关闭煤矿24处，52家烟花爆竹企业全部退出。食品药品安全形势持续向好。扎实推进基层治理网格化、综治中心实体化，社会治理水平不断提升。加大力度化解信访问题，赴省访、来市访人次分别下降23.4%、33%。扫黑除恶专项斗争深入开展，全国禁毒示范城市创建工作成效明显，刑事警情下降18.4%，八类案件发案下降58.4%，人民群众安全感进一步提升。

（五）坚持对标对表，自身建设从严从实

始终坚持正确政治方向，牢固树立“四个意识”，坚决做到“两个维护”，全面落实从严治党要求。推进法治政府建设，修订完善《市政府工作规则》，落实常务会议专题学法和邀请法律顾问列席等制度。自觉接受人大法律监督、工作监督和政协民主监督，认真办结人大代表建议99件、政协提案192件。在全市政府系统开展了忠诚型、创新型、担当型、服务型、过硬型“五型”政府建设，坚决整治“怕慢假庸散”等作风顽疾，大力倡导“事事马上办、人人钉钉子、个个敢担当”，着力打造“四最”营商环境，干部作风和精神状态持续好转。严格落实中央八项规定精神，坚决全面彻底肃清苏荣案余毒，扎实推进省委巡视反馈问题整改，严肃查办违纪违法案件，反腐败斗争压倒性态势持续巩固发展。

此外，民族宗教、妇女儿童、青少年、国防动员、科学普及、社科研究、红十字、老龄、慈善等工作都取得新成绩。

各位代表！过去一年所取得的成绩和进步，是习近平新时代中国特色社会主义思想科学指引的结果，是市委团结带领全市人民奋力拼搏的结果。在此，我代表市人民政府，向全市人民，向各民主党派、人民团体和社会各界人士，向驻饶部队、武警部队官兵，向中央和省驻饶单位，以及所有关心、支持上饶发展的同志们、朋友们，表示衷心的感谢！

各位代表！回首2018年，挑战无处不在，历程充满艰辛，成绩来之不易。我们深深体会到：唯有不忘初心、牢记使命，坚持党的领导，紧紧依靠全市人民，才能办好上饶的事情；唯有坚持以人民为中心的发展思想，维护群众利益，不断满足人民日益增长的美好生活需要，才能赢得信任和支持；唯有稳扎稳打、埋头苦干，努力比别人干得更多、干得更实、干得更拼，才能实现科学发展、跨越赶超。实践再次证明，任何艰难险阻都挡不住上饶人民坚毅前行的步伐。面对未来，我们有信心、有决心、有条件，更有办法不断创造新的更大辉煌！

同时，我们也清醒看到，我市经济社会发展中还存在不少困难和问题。主要是：发展不平衡不充分问题仍然突出，产业“低小散”格局还没有根本改变；创新驱动支撑不足，新旧动能转换还不够快；优质公共服务供给不足，教育、医疗等民生领域还存在不少短板，脱贫攻坚、污染防治任务依然艰巨；少数工作人员缺乏担当精神，一些领域不正之风还不同程度存在。我们一定高度重视这些问题，采取有力措施，切实加以解决。

二、奋力冲、拼力跑，跑好高质量跨越式发展的接力赛

2019年是新中国成立70周年，是决胜全面小康、打造大美上饶的关键之年。做好今年工作，意义十分重大。今年政府工作的总体要求是：以习近平新时代中国特色社会主义思想为指导，深入贯彻党的十九大、十九届二中、三中全会、中央经济工作会议、省委十四届七次全会和市委四届七次全会精神，从更高层次贯彻落实习近平总书记对江西工作的重要要求，坚持稳中求进工作总基调，坚持新发展理念，坚持高质量跨越式发展首要战略，坚持以供给侧结构性改革为主线，坚持深化市场化改革、扩大高水平开放，统筹做好产业升级、城乡建设、改革开放、县域发展、民生保障、社会治理等工作，切实巩固上饶来之不易的好势头、好局面、好氛围，努力保持经济持续健康发展和社会大局稳定，为与全国全省同步全面建成小康社会收官打下决定性基础，奋力迈出“决胜全面小康、打造大美上饶”的坚实步伐，以优异成绩庆祝新中国成立70周年。

主要预期目标是：生产总值增长8.2—8.6%，财政总收入增长6%，规模以上工业增加值增长8.6%左右，固定资产投资增长9.2%左右，社会消费品零售总额增长10.6%左右，实际利用外资增长6.1%，城镇居民人均可支配收入增长8.5%，农村居民人均可支配收入增长9%，居民消费价格总水平涨幅控制在3%左右，城镇登记失业率控制在4.5%以内，节能减排完成省下达任务。

各位代表！习近平总书记指出，“对定下来的工作部署，要一抓到底、善始善终。要有‘功成不必在我’的境界，像接力赛一样，一棒一棒接着干下去。”高质量跨越式发展是省委十四届六次全会确立的首要战略，是当前和今后一个时期的接力赛，需要我们一年接着一年、一棒接着一棒跑下去。今年，我们将紧扣既定目标，努力在七个方面跑出好成绩。

（一）努力跑出主攻工业的好成绩

持之以恒抓好产业、企业、项目三大关键要素，推动工业经济总量和质量同步提升。

加速壮大主导产业。全面落实《工业崛起“2050”工程实施意见》，开辟“绿色通道”，强化资金、土地、用工等要素支撑，确保年内上饶经开区至少引进一个投资超50亿元的产业项目，各省级开发区至少引进一个投资超20亿元的产业项目。深入推进上饶经开区世界光伏城、中国光学城、江西汽车城“三城”创建，继续实施晶科能源“双倍增”项目，确保海康智慧物联网产业园、维真裸眼3D项目建成投产，中汽瑞华、博能商用车整车下线。大力开展“县域工业发展巩固年”活动，坚定不移培育壮大主导产业，致力打造有色、光伏、汽车等3个千亿级产业，电子信息、新型建材、现代纺织等3个五百亿级产业，光学、食品等若干个百亿级产业，引领带动全市产业上规模、提层次、增效益。

持续做强园区平台。深化园区管理体制改革，建立全链审批赋权清单，推动县级经济管理权限向园区“下移”，完善“与发展成效相匹配、与工作业绩相挂钩”的人事和薪酬制度，全面增强园区发展内生动力，让工业园区真正成为“大家都想去、去了能干事、干事能成事”的好平台。实施“两型三化”园区管理提标提档行动，开展智慧园区创建，推动园区开发建设、运营管理和服务水平全面提升，年内实现“园区事园区内办结”。树立“以亩产论英雄”导向，强化“两率一度”考核，加快处置“僵尸企业”“僵尸项目”，有效盘活闲置土地，提升集约节约用地水平。

创新工业发展模式。运用“产业集聚、产城融合、产融互动、资源共享”理念，推进小微产业园建设，协同破解工业发展“招商难”“用地难”“融资难”“监管难”等实际问题。高起点、高标准规划建设信江产业新城，力争引进温州万洋集团等一批龙头项目“开篇破题”，致力打造上铅快速通道“黄金工业走廊”。统筹推进标准厂房建设和项目引进，加速企业“拎包入驻”、产业“延链壮链”。

加大企业帮扶力度。围绕“兴实业、强实体”，持续“减负担、增效益”，精准推进降成本优环境专项行动，积极跟进国家减税降费举措，加快涉企行政事业性收费“清零”。完善市县领导挂点帮扶企业制度，加大援企稳岗力度，竭力为企业减轻负担、排忧解难。

各位代表，工业是国民经济的“脊梁”。我们必须坚持“主攻工业、决战园区”不动摇，把工业发展作为第一位经济工作，努力让产业层次高起来，让企业实力强起来，让项目建设快起来，不断夯实高质量跨越式发展的基础支撑。

（二）努力跑出现代服务业发展的好成绩

坚持扬优挖潜，聚焦做大规模、做响品牌，不断提升现代服务业发展能级。

加速做大数字经济。完成大数据科创城规划编制，推进大数据创新应用园区、数字文化应用园区、上饶呼叫城、文娱创意中心二期、游戏产业孵化中心、电竞总部基地、中科数创园、大数据创新创业中心等平台建设，力促企业轻装落户、快速集聚。依托华为、滴滴、贪玩等龙头企业，引进更多上下游关联性重大项目。推动全省电子政务外网数据备份中心、全省旅游数据中心、全省交通数据备份中心尽早落户。全面落实产业扶持政策，统筹实施包容审慎监管，严格执行全程代办制度，为新业态成长创造宽松环境，真正让数字经济在上饶生根扎根、开花结果。

加速做强全域旅游。推进城东文化旅游综合体、“十里槠溪”国际生态旅游度假区、捷成梦都影视城、野生动物乐园、灵山光影小镇、法国PVCP集团乡村度假旅游综合体等项目，加快补齐城区旅游短板。依托众多秀美乡村点，加快完善游乐设施，健全车位、餐位、床位、厕位、摊位等功能配套，打造一批精品线路，出台乡村旅游标准化服务规范，办好全国乡村旅游发展经验交流现场会，继续举办乡村旅游文化节，促进乡村旅游持续升温。推进篁岭景区创5A、灵山大峡谷等景区创4A工作，进一步提升景区发展水平。大力促进文旅融合，推进信江文化经济带建设，开展“可爱的中国”文化旅游年主题活动。积极创建全国旅游标准化试点城市，致力打造旅游目的地、中转地、集散地。

加速做实大健康产业。发挥上饶国际医疗旅游先行区、国家中医药健康旅游示范区两大平台作用，紧扣“医、养、健”主题，强化规划引领，推进医旅融合，大力引进一批具有示范、带动、引领作用的龙头企业、骨干项目，尽快展现核心区实物形象。加速恒大国际医院和养生谷、江西汉氏联合医院、上饶国际精准医疗中心、上饶旅游健康体验园等项目建设，致力推动先行区升格为国家级。实施道地药材种植扩面、知名药企入饶、饶信医药振兴“三大工程”，加快德兴中国中医科学院试验培训基地、横峰药用植物园建设，努力打造中医药健康旅游的“上饶标准”。

加速做活商贸、物流、金融业。实施促进商贸消费升级三年行动，强化优品、兴市、强商、旺客、捷运五大举措，进一步激发消费潜力，提升消费水平，让消费者放心消费、乐享消费。办好第三届饶帮菜美食文化节。加快建设上饶国际物流园、江天农产品冷链物流园二期等项目。持续实施“映山红行动”，力争年内新增主板上市企业2家以上，全市直接融资额增长20%以上。

各位代表，服务业是新旧动能转换的“主引擎”，是就业的最大“容纳器”。我们必须抢抓机遇、抢占风口，深耕现代服务业沃土，厚植新兴服务业幼苗，推动服务业茁壮成长、持续领先。

（三）努力跑出宜居城市建设的好成绩

深入推进城市功能与品质提升三年行动，全面实施城市治脏、治乱、治堵、功能修补、生态修复、彰显特色、亮化美化、治理创新“八大行动”，实现“一年有提升、两年上台阶、三年进一流”。

提升综合交通枢纽功能。推进对外交通畅通工程，启动三清山机场扩建、德兴至景德镇高速公路、金华—衢州—上饶高速公路、杭州（富阳）至上饶（德兴）高速公路前期工作，支持昌景黄高铁、德州至上饶高速公路建设，加速上饶动车组存车场、上浦高速公路、鄱余高等级公路等项目建设。推进城区交通畅通工程，新建富饶路东延伸、融创路、吴芮路、溪畔路等12条道路，新增城市道路25公里。

提升城市承载能力。以市民需求为导向，强化市政基础、公共安全等设施规划建设和管控，补齐功能短板。建成城北、城南公交枢纽总站，建设公交首末站23个，购置新能源公交车200标台。加快地下综合管廊和海绵城市建设。优化布局教育、医疗、文化、体育等公共服务设施。持续推进棚户区改造和公共租赁住房建设。新建标准化菜市场5个，改造提升9个。把里弄小巷、老旧小区作为感知群众冷暖的“温度计”，全面排查摸底，分步改造提升，年内实施里弄小巷改造20条、老旧小区改造10个。

提升城市管理水平。坚持以市民为主体的创建理念，发动全员参与，深入开展全国文明城市和国家卫生城市“双城”同创，使文明卫生渗入城市肌理、融入百姓生活。推行“以克论净、深度保洁”环卫作业规范，推进“道路本色、立面清爽”行动，确保中心城区道路机扫率达到85%以上，其他县（市）达到65%以上。规范店招门楣和广告牌设置，启动城市架空管线整治。加快交通管理智能化建设，推进城区治堵提速。

各位代表，城市让生活更美好。我们必须顺应城市加速转型发展的趋势和规律，抓紧完善功能，不断提升品质，努力把上饶打造成为令创业者称赞、本地人自豪、外地人向往的现代化城市。

（四）努力跑出乡村振兴的好成绩

围绕农业全面升级、农村全面进步、农民全面发展，深入实施乡村振兴战略，推动农业农村现代化。

让农业产业“旺”起来。深入落实农业结构调整“十大工程”和“东西南北中”特色产业布局，大力发展品牌农业、规模农业、工厂农业、智慧农业，推进农业标准化、机械化、优质化，着力打造马家柚、虾蟹、茶叶三个百亿级产业。加快建设农产品加工产业园，提高农副产品就地转化率。推进“菜篮子”工程建设，年内新增蔬菜种植面积10万亩。建设高标准农田54.6万亩。

让农村要素“活”起来。巩固和完善农村基本经营制度，深化农村承包地“三权分置”改革。抓好农村集体产权制度改革，持续壮大村集体经济，培育一批经济强村。盘活闲置宅基地、农村空闲地、工矿废弃地等资源，推进一批城乡建设用地“增减挂钩”项目。大力实施新型职业农民培育工程和“一村一名大学生”工程，让各类人才在广阔农村尽展才华。

让乡村环境“美”起来。新建秀美乡村点5043个，力争提前一年基本完成“扫一遍”任务。推进“四好农村路”创建工作，新改建农村公路1000公里，改造危桥100座。升级改造农村电网264公里。鼓励有条件的行政村或较大自然村因地制宜建设污水处理设施。严格执行《农村居民住房建设管理条例》，全面落实“一户一宅”制度。加强农村风貌管控，彰显特色，传承乡愁。继续推进公益性墓地建设，巩固绿色殡葬改革成果，确保火化率、入公墓率两个100%。深化农村人居环境整治，不断改善乡村环境面貌。

让农民生活“好”起来。拓宽农民增收渠道，确保农村居民收入稳定增长。积极实施乡村就业创业促进行动，带动更多农民在家门口就业创业。将法治与德治相结合，实施“法律明白人”培养工程，传承优秀传统文化，大力塑造文明乡风、良好家风、淳朴民风，让广大农民既富“口袋”、又富“脑袋”。

各位代表，农业农村农民问题是关系国计民生的根本性问题，“三农”是“压舱石”和战略后院。我们必须始终坚持农业农村优先发展，统筹落实乡村振兴重点任务，努力绘就大美上饶秀美乡村新画卷。

（五）努力跑出改革开放的好成绩

紧盯问题抓改革，紧盯产业扩开放，紧盯实效谋创新，不断激发高质量跨越式发展的动力活力。

抓好重点领域改革。全面完成市县机构改革。深化供给侧结构性改革，抓紧淘汰落后产能，关闭煤矿12处。深化上投集团投资运营体制改革，不断提升市场竞争力和服务经济社会发展能力，力争集团资产总额达到2500亿元、营业收入达到100亿元。稳妥处置地方政府债务风险，加大防范处置非法集资力度，构建房地产市场健康发展长效机制，坚决打好防范化解重大风险攻坚战。持续推进政务服务“一网、一门、一次”改革，一切从方便群众和企业办事出发，深入开展“减证便民”行动，全面推行电子证照应用，市本级政务服务大厅确保在5月份全面升级运行，年底前县（市、区）依申请类政务服务事项“一次不跑”“只跑一次”办理率达到70%以上。深化商事制度改革，放大“多证合一”改革效应，全面推广“证照分离”，实现“照后减证”，提升市场准入便利化程度。构建生产、流通、消费全链条新型市场监管机制，全面推行“双随机一公开”监管，释放市场活力，保障消费安全。

提升开放合作水平。对标江西开放门户城市定位，发挥高铁十字交汇优势，聚焦交通、产业、公共服务等重点领域，从更高层次全面对接、融入长三角区域一体化发展。支持鄱余万融入大南昌经济圈。推进浙赣边际合作衢饶示范区建设。以“三请三回”、“三企入饶”、主导产业招大引强活动为抓手，大力引进一批龙头项目、骨干项目，进一步引导饶商资金回归、技术回归、总部回归、人才回归。完善抓洽谈签约、抓开工竣工、抓核查巡查、抓分析调度“四抓”工作机制，提升招商引资实效。

增强创新驱动能力。着力构建以企业为主体、市场为导向、“产学研用金”相结合的技术创新体系。深入实施创新型企业、高新技术企业和科技型中小企业培育工程，引导各类创新要素向企业聚集。推进上饶（国际）基金双创生态示范城建设。争创上饶国家新能源汽车高新技术产业化基地。提升横峰江西绿色生态葛研究所创新实力。完善高层次人才引进方式，健全柔性引才机制，打好招才引智“组合拳”，形成聚才留才“强磁场”。深入实施质量强市战略，全面提高产品、环境、工程、服务领域质量水平。

大力发展民营经济。全面落实鼓励民营经济发展各项措施，坚决让政策落地生根、让红利有效释放、让企业得到实惠。打破各种“卷帘门”“玻璃门”“旋转门”，让民营经济创新源泉充分涌流、创造活力充分迸发。积极构建亲清新型政商关系，多做“雪中送炭”的好事、“排忧解难”的实事，做到周到服务、热情服务、温馨服务。依法保护民营企业家人身安全和财产安全，让民营企业家在上饶投资放心、发展安心、生活舒心。

各位代表，改革开放是决定当代中国命运的关键一招。我们必须倍加珍惜40年积累的宝贵经验，将改革开放进行到底，将创新引领坚持到底，让市场化改革、高水平开放成为新时代上饶的新标签。

（六）努力跑出生态文明建设的好成绩

坚决落实生态优先、绿色发展理念，努力打通绿水青山与金山银山双向转换通道，进一步擦亮上饶绿色生态“金字招牌”。

打好污染防治攻坚战。盯紧抓实中央和省环保督察问题整改，深入推进长江经济带“共抓大保护”攻坚行动。持续开展净空净水净土行动，深化工地和道路扬尘、餐饮油烟、工业废气、机动车尾气等专项治理，巩固城市建成区禁放烟花爆竹工作成果，让“上饶蓝”成为常态。深入推进以鄱阳湖为主的河湖全流域治理。认真实施《大坳水库饮用水水源保护条例》，启动大坳水库生活污水治理项目。力争年内新增污水管网160公里以上。促进农药化肥减量使用，加强固体废弃物处置管控，不断改善土壤环境质量。持续打好环保问题治理的“人民战争”，严厉打击环境违法犯罪行为。

加大生态修复与建设力度。坚持“减少存量、严控增量”，推动企业减量排放，杜绝“三高一低”项目。加强山体保护、地质灾害防治和水土流失综合治理。实施森林质量提升工程，完成植树造林8.3万亩。全面铺开生活垃圾分类试点，加速中心城区、弋阳、婺源、德兴、鄱阳、余干等地生活垃圾焚烧发电项目建设，推进全市城乡生活垃圾处理无害化。

健全和落实生态文明制度。严格执行永久基本农田保护红线，统筹生态保护红线和城市开发边界划定工作。深入实施河长制、湖长制、林长制。全面推行领导干部自然资源资产离任审计。推进公共机构节能，推动绿色低碳的生产生活方式在全社会深入人心。

各位代表，环境就是民生，青山就是美丽，蓝天也是幸福。我们必须坚决摒弃“先污染、后治理”的老路，倍加呵护上饶这一方碧水蓝天。对于那些有环境污染的项目，哪怕有再大的投资，我们也坚决不要；哪怕能产生再高的GDP，我们也坚决不要；哪怕会带来再多的税收，我们也坚决不要。

（七）努力跑出增进民生福祉的好成绩

自觉践行以人民为中心的发展思想，紧扣群众关切，尽力而为、量力而行，守住底线、筑牢底部，不断满足人民日益增长的美好生活需要。

打好脱贫攻坚战。坚持问题导向，创新工作举措，狠抓教育、医疗、住房“三保障”政策落实，确保高质量完成1个贫困县摘帽、67个贫困村退出、8.3242万贫困人口脱贫的年度任务。坚持“输血”“造血”双管齐下，重点推进产业扶贫长效化、精神扶贫实效化，精心搭建产业与贫困户、村集体的利益联结机制，组织开展脱贫攻坚“感恩行动”，大力营造勤劳致富、脱贫光荣的浓厚氛围。统筹推进贫困县与非贫困县、城镇与农村贫困人口脱贫，建立健全稳定脱贫长效机制，确保遇困、遇病、遇灾不返贫。

推进社会事业繁荣发展。深入实施第三期学前教育行动计划，推进乡镇示范性公办幼儿园建设。开工建设槠溪学校、陆羽小学，启动市一中、二中原教师宿舍棚户区改造，确保市一中新校区、三中、信美学校、十五小、时乔小学秋季开学，着力化解“大班额”“大校额”问题。实施名师名校长领航工程，深化与华师大、北师大、北大教育学院等名校合作办学。推进高水平院校、高水平专业“双高”计划实施。支持上饶师院申请硕士点。支持江西医专创建应用型本科医学院校。筹建江西汉氏生命科技学院。积极推进医联体和远程医疗体系建设。抓好德兴分级诊疗一体化改革试点工作。开工建设120紧急救援中心和皮肤病医院。举办方志敏诞辰120周年纪念活动。办好上饶第三届文博会。大力弘扬万年稻作文化、千年书院文化、百年红色文化等地方特色文化，不断扩大饶信文化影响力。推进新时代文明实践中心建设，提升文化馆、图书馆、博物馆和镇村综合性文化服务中心效能，努力使群众文化生活就近就便、多姿多彩。完善体育设施共建共享机制，启动健身步道建设，广泛开展全民健身活动，办好上饶国际马拉松赛、玉山中式台球世锦赛等品牌赛事。

织密扎牢民生保障网。落实就业优先战略，重点抓好高校毕业生、退役军人、返乡创业人员等群体的就业创业服务。深化全民参保计划，对中小企业职工、农民工、灵活就业人员、新业态从业人员等重点人群实施精准扩面，完善企业职工养老保险制度，进一步提高城乡居民基本养老、基本医保和低保等补助标准。大力发展社会救助、社会福利、公益慈善等事业。适应人口老龄化趋势，支持居家和社区养老服务发展。

加强和创新社会治理。加大“雪亮工程”建设力度，不断完善“社情民意、网格收集，网格发声、综治响应，综治吹哨、部门报到”的综治新模式，有效预防和化解各类矛盾纠纷。依法严厉打击违法犯罪活动，坚持有黑必扫、有恶必除、有伞必打、有腐必反、有乱必治，坚决铲除黑恶势力滋生土壤。巩固禁毒示范城市创建工作成果。加强信访工作与调解、仲裁、行政裁决、行政复议等衔接配合，维护良好信访秩序。加强社会信用体系建设，营造诚实守信的社会风尚。完善应急管理和防灾减灾救灾体制机制，切实保障人民群众生命财产安全。

各位代表，人民是国家的主人，民心是最大的政治。我们必须把人民利益摆在至高无上的地位，始终同人民想在一起、干在一起，努力让发展更有“温度”、让幸福更有“质感”。

各位代表！跑好今年的接力赛，需要全市政府系统进一步强身健体、提质塑能。我们将持续推进“五型”政府建设，不断增强政府公信力和执行力。一是始终做到忠诚真诚。坚持忠诚于党，把党的领导作为最高政治原则，牢固树立“四个意识”，坚决做到“两个维护”，全面落实党中央、国务院、省委、省政府和市委决策部署。坚持真诚为民，恪守人民至上的价值取向，聚焦群众关切，攻坚民生短板，让所有工作都充分体现人民意愿、维护人民利益、接受人民监督。二是始终做到尊法守法。严格遵守宪法法律，严格执行重大行政决策程序规定，严格依法行政，坚决做到“法无授权不可为、法定职责必须为”。认真落实市人大及其常委会各项决议决定，积极支持政协履行职能，及时办理人大代表议案建议和政协提案，自觉接受人大依法监督、政协民主监督以及监察、司法、审计和社会监督，全力提高履职科学化水平。三是始终做到懂行在行。各级政府工作人员都要加强学习钻研，努力做到对政策了如指掌、对情况如数家珍，成为所负责工作的“行家里手”。健全“问题导向+解决方案”推进机制，瞄准影响经济社会发展的全局性问题、制约转型升级的突出矛盾、群众反映强烈的民生诉求，研究制定精准措施，务实高效破解发展难题。四是始终做到苦干实干。以“干事为荣、避事为耻”，把干事创业谋发展作为最大责任，勇于挑最重的担子，敢于啃最硬的骨头，善于接最烫的山芋。健全正向激励和容错纠错机制，完善执行、监督、考核、奖惩机制，确保事事雷厉风行马上办、人人锲而不舍钉钉子、个个义不容辞敢担当。五是始终做到勤政廉政。保持“一日无为、三日不安”的精神状态，坚决克服懒政、怠政，让“不为不办找理由、只为办好想办法”成为全市各级政府及其工作人员的鲜明标识。牢固树立过“紧日子”思想，大力压缩一般性支出。强化反腐倡廉，对违规违纪违法，踩“红线”、越“底线”、闯“雷区”的，不以权势大而回避，不以问题小而姑息，不以违者众而放任。持续加强作风建设，坚决反对形式主义、官僚主义，严厉整治“怕慢假庸散”作风顽疾，不断涵养政府系统清风正气。

各位代表！习近平总书记在新年贺词中指出：“2019年，有机遇也有挑战，大家还要一起拼搏，一起奋斗。”让我们更加紧密地团结在以习近平同志为核心的党中央周围，在省委、省政府和市委的坚强领导下，锐意进取，扎实工作，以优异成绩庆祝新中国成立70周年，为“决胜全面小康、打造大美上饶”、谱写高质量跨越式发展新篇章而接续拼搏、不懈奋斗！
